# Anemia and red blood cell transfusion in critically ill cardiac patients

**DOI:** 10.1186/2110-5820-4-16

**Published:** 2014-06-02

**Authors:** Geneviève Du Pont-Thibodeau, Karen Harrington, Jacques Lacroix

**Affiliations:** 1 Sainte-Justine Hospital, Room 3431, 3175 Côte Sainte-Catherine, Montreal, QC H3T 1C5, Canada

**Keywords:** Blood, Cardiac, Critical care, Erythrocyte, Hemoglobin, Intensive care, Practice, Risk factors, Surgery, Transfusion

## Abstract

Anemia and red blood cell (RBC) transfusion occur frequently in hospitalized patients with cardiac disease. In this narrative review, we report the epidemiology of anemia and RBC transfusion in hospitalized adults and children (excluding premature neonates) with cardiac disease, and on the outcome of anemic and transfused cardiac patients. Both anemia and RBC transfusion are common in cardiac patients, and both are associated with mortality. RBC transfusion is the only way to rapidly treat severe anemia, but is not completely safe. In addition to hemoglobin (Hb) concentration, the determinant(s) that should drive a practitioner to prescribe a RBC transfusion to cardiac patients are currently unclear. In stable acyanotic cardiac patients, Hb level above 70 g/L in children and above 70 to 80 g/L in adults appears safe. In cyanotic children, Hb level above 90 g/L appears safe. The appropriate threshold Hb level for unstable cardiac patients and for children younger than 28 days is unknown. The optimal transfusion strategy in cardiac patients is not well characterized. The threshold at which the risk of anemia outweighs the risk of transfusion is not known. More studies are needed to determine when RBC transfusion is indicated in hospitalized patients with cardiac disease.

## Review

### Introduction

Red blood cell (RBC) transfusion is common in critically ill adults and children [1-3]. Patients with cardiac disease are transfused at higher hemoglobin (Hb) thresholds than those with non-cardiac illness [1-3]. Both anemia and RBC transfusion are associated with increased mortality in cardiac patients. The risk/benefit balance of RBC transfusion in critically ill cardiac patients is currently a matter of debate.

This narrative review critically appraises the available data on the relationship between anemia, RBC transfusion and outcome of critically ill adults and children with cardiac disease. We discuss the prevalence and risks of anemia in the ICU, the rationale for RBC transfusion, the evidence supporting restrictive transfusion strategies, and the growing interest in goal-directed transfusion therapy. Premature infants and intraoperative transfusions will not be addressed in this paper, nor will a detailed discussion of the relationship between length of storage of RBC units and outcomes, since the latter is discussed in other papers [4-7].

### Anemia

**Figure 1:**
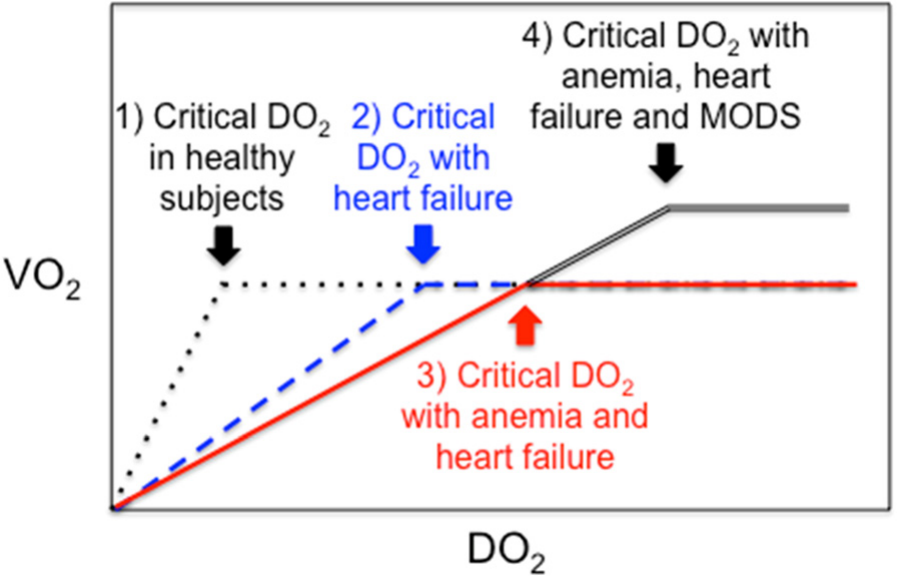
**Critical oxygen delivery (DO**_**2**_**), cardiac failure, anemia and multiple organ dysfunction syndrome (MODS).** X axis:systemic DO2; Y axis: global oxygen consumption (VO_2_). Critical DO2: level below which DO_2_ does not meet oxygen demand (O_2_ supply dependency). (1) In healthy subjects (dotted black and white line), as DO_2_ decreases, VO2 remains constant by compensatory mechanisms (increased cardiac output and cellular O_2_ extraction). (2) Cardiac failure limits compensatory increase in cardiac output (hatched blueline). (3) This limitation is worst in patients with anemia and heart failure (plain red line) because low hemoglobin decreases arterial oxygen content (CaO_2_). (4) Severe cardiac dysfunction may be associated with systemic inflammatory response syndrome (SIRS) and multiple organ dysfunction syndrome (MODS), both of which increase systemic VO_2_ and therefore critical DO_2_ (double black line).

The prevalence of anemia in adults with heart failure ranges from 18 to 38% [8]; it was 19.1% in 27 observational studies indexed before July 2011 that enrolled 233,144 patients with acute coronary syndrome [9]. Anemia may result in insufficient oxygen delivery (DO_2_) to vital organs and tissues if DO_2_ drops below a critical DO_2_ (Figure 1). DO_2_ is determined by cardiac output (Q’) and arterial content in:

DO_2_ = Q’ × CaO_2_

and CaO_2_ = {(Hb × SaO_2_ × 1.39) + (PaO_2_ × 0.0031)} [10]

Any drop of the Hb level decreases CaO_2_ and DO_2_ if the compensatory increase of Q’ is not high enough. Anemia may be poorly tolerated by patients with cardiac failure and/or coronary disease because their ability to increase cardiac output to compensate for anemia is limited [11,12].

**Figure 2:**
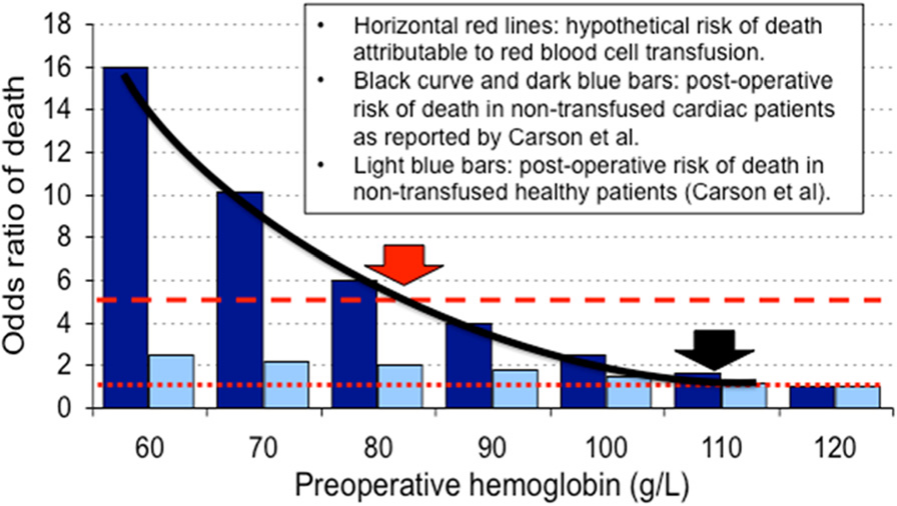
**Cost-benefit analysis of red blood cell (RBC) transfusion.** The background histogram is drawn from data published by Carson *et al*. [13] (with permission) illustrating the relationship between pre-operative anemia and surgical mortality in 1,958 Jehovah’s Witness patients. Dark blue bars: preoperative hemoglobin (Hb) versus odds ratio (OR) of death in cardiac patients (risk of death increases as Hb level falls). Light blue bars: pre-Hb versus OR of death in patients who were healthy before surgery. We draw on this histogram: (1) dotted horizontal red line: no risk of mortality attributable to RBC transfusion (OR = 1); (2) hatched horizontal red line: hypothetical higher risk of mortality attributable to RBC transfusion (OR = 5); (3) plain curved black line: pre-operative Hb versus odds ratio (OR) of death in cardiac patients. A RBC transfusion is probably more useful than harmful when Hb level is below the intersection between the black curve describing the risk of mortality associated with anemia and the red line describing the risk of mortality attributable to RBC transfusion. If RBC transfusions are perfectly safe (OR = 1), the dotted horizontal red line and the black curve cross together at a Hb level of about 110 g/L (black arrow). If the risk of mortality attributable to RBC transfusion is high (for example, OR = 5), the hatched horizontal red line and the black curve cross together at a Hb level of about 80 g/L (red arrow). Where the true curve lies for cardiac patients is unknown. The curved line would probably move to the right in severely ill cardiac patients, and to the left if cardiac dysfunction is milder. The actual risk of death attributable to RBC transfusions in different cardiac populations remains to be determined.

Carson *et al*. [13] studied the association between anemia and surgical mortality in 1,958 Jehovah’s Witness patients with cardiovascular disease who refused transfusion; the risk of mortality was inversely related to Hb level (Figure 2). Table 1 summarizes the results of two meta-analyses [9,14] and six observational studies [15-20] on the relationship in cardiac adults between anemia and adverse outcomes; a statistically significant association is reported in almost each instance. However, it must be underlined that the relationship between anemia and outcome is fundamentally confounded in all these studies by co-morbidities that might have caused the anemia (renal failure, gastro-intestinal bleeding, iron deficiency, and so on).

**Table 1:** Anemia and outcomes in cardiac adults: observations studies

	**Health problem**	**Patients**	**Studies**	**Risk**
Outcome		(n)	(n)	(95% CI)
Meta-analyses				
Mortality^a^[14]	ACS	51,449	10	aOR: 1.49 (1.24 to 1.79)
Mortality^b^[9]	ACS	171,915	27	aHR: 1.49 (1.23 to 1.81)
Studies not included in the meta-analyses				
Mortality [15]	New heart failure	12,065	1	aHR: 1.34 (1.24 to 1.46)
Mortality [16]	PCI	6,116	1	aHR: 1.8 (1.3 to 2.3)
Mortality [17]	PCI	48,851	1	aOR: 2.29 (1.79 to 2.92)
Mortality [18]	Cardiac surgery	13,843	1	12.5% versus 7.5%, *P* = 0.014
Mortality [19]	CABG	2,102	1	aOR: 0.99 (0.98 to 1.0)
Mortality [20]	ACS	7,922	1	aOR: 1.71 (1.34 to 2.17)
Meta-analyses on other outcomes				
Heart failure^a^[14]	ACS	152,849	5	OR: 1.96 (1.47 to 2.62)
Cardiogenic shock^a^[14]	ACS	129,136	4	OR: 1.95 (1.04 to 2.64)
Reinfarction^b^[9]	ACS	22,115	6	RR: 1.25 (1.02 to 1.53)

ACS: acute coronary syndrome; aHR: adjusted HR; aOR: adjusted odds ratio; CABG: coronary artery bypass graft; CI: confidence interval; HR: hazard ratio; OR: odds ratio; PCI: percutaneous coronary intervention; RR: relative risk.

This table reports data from two meta-analyses [9,14] and six observational studies [15-20] that were not included in these meta-analyses.

^a^Overall, the meta analysis conducted by Liu *et al*. [14] included 241,293 patients enrolled in 19 studies; the analysis on mortality (10 studies) disclosed a very important heterogeneity (*I*^*2*^ = 84%).

^b^Overall, the meta analysis conducted by Lawler *et al*. [9] included 233,144 patients. The analyses on mortality and reinfarction included 27 and 10 studies respectively; an important heterogeneity was found in both instances (*I*^*2*^ = 98.2% and 48.4% respectively).

A number of host characteristics specific to children, like growth, fetal Hb, different cardiac diseases and physiology, may impair their adaptive mechanisms to anemia. There are few data on the relationship between anemia and outcomes in cardiac children. Kammache *et al*. [21] reported some anemia (Hb < 100 g/L) in 24% of 218 children with idiopathic dilated cardiomyopathy; mortality was more frequent in anemic children.

### Transfusion

In the United States, 7.8% to 92.8% of adults undergoing cardiac surgery are transfused [22-24]. A transfusion is given after cardiac surgery in 38% to 74% of children [3,25-28].

RBC transfusions are sometimes given to cardiac patients to rapidly replace blood volume in cases of postoperative blood losses [29]. In the absence of active bleeding, some practitioners declare that low Hb concentration and/or signs of inadequate DO_2_ (low central venous O_2_ saturation (ScvO_2_), high lactate level, low SaO_2_/FiO_2_ ratio) would prompt them to prescribe RBC transfusions in this population [29]. There is no doubt that Hb level is a very important determinant of RBC transfusions in critically ill patients [30-32]. However, there are data suggesting that Hb level is not the only determinant of RBC transfusions in cardiac patients. In a retrospective study on transfusions in pediatric cardiac ICU [24], admission Hb ranged from 141 to 150 g/L, nadir Hb level was 121 g/L in patients who were not transfused, 119 g/L in a low transfusion group, and 115 g/L in a high transfusion group, suggesting that transfusions may have been given for reasons other than anemia. Age below one year, low weight, high severity of illness as measured by the acute physiology and chronic health evaluation (APACHE) or pediatric risk of mortality (PRISM) score, cardiopulmonary bypass, cyanotic heart condition and lower admission Hb level are also reported to be independently associated with increased administration of RBC transfusions [24,33].

## Risks and benefits of RBC transfusions

### Non-cardiac patients

RBC transfusion should be given only when the risk/benefit ratio is favorable (ratio < 1). The threshold at which the risks of anemia outweigh the risks of transfusion is currently unknown.

There are little available hard data on the clinical benefits of RBC transfusion. In a prospective study that enrolled 303 Kenyan children with Hb below 50 g/L at hospitalization, 116 (38%) did not receive a transfusion, mostly because of blood unavailability, while 187 did; the mortality was significantly higher in non-transfused children (41.4% versus 21.4%, *P* < 0.001) [34]. This study suggests that RBC transfusion may improve the survival of anemic hospitalized patients with Hb level below 50 g/L, but what threshold above 50 g/L should be used to prescribe a RBC transfusion in cardiac patients remains a matter of debate.

### Adult cardiac patients

The available evidence suggests that RBC transfusion in patients with cardiac disease is an independent risk factor of mortality (Table 2).

**Table 2:** **Red blood cell (RBC) transfusions and outcomes in cardiac patients: observational studies**^**a**^

	**Health problem**	**Patients**	**Risk**^**c**^	
**Outcome, first author, year**^**b**^		**(n)**	**(95% CI)**	***P-*** **value**
Outcome: mortality in adults				
Mortality, Alexander, 2008 [35]	ACS	44,242	OR: 3.2 (2.9 to 3.6)	
	ACS and Hct ≤ 24%		aOR: 0.68 (0.45 to 1.02)	
	ACS and Hct = 24 to 27%		aOR: 1.01 (0.79 to 1.30)	
	ACS and Hct = 27 to 30%		aOR: 1.18 (0.92 to 1.50)	
	ACS and Hct > 30%		aOR: 3.47 (2.30 to 5.23)	
Mortality, Aronson, 2008 [36]	AMI	2,358	aHR: 0.13 (0.03 to 0.65)^d,e^	0.013
			aHR: 2.2 (1.5 to 3.3)^d,f^	< 0.0001
Mortality, Jani, 2007 [37]	AMI	4,623	aOR: 2.02 (1.47 to 2.79)	< 0.0001
			RR: 4.83 (3.81 to 6.12)^d^	
Mortality, Jolicœur, 2009 [38]	AMI	5,188	RR: 6.38 (4.88 to 8.34)	
		5,532	aHR: 2.16 (1.20 to 3.88)	< 0.0001
		5,188	RR: 6.38 (4.88 to 8.34)^d^	
Mortality, Koch, 2006 [39]	CABG	5,814	OR: 1.77 (1.67 to 1.87)	< 0.0001
Mortality, Murphy, 2007 [40]	Cardiac surgery (UK)	8,518	HR: 6.69 (3.66 to 15.1)	< 0.05
Mortality, Nikolsky, 2009 [41]	AMI	2,060	HR: 4.71 (1.97 to 11.36)	
			RR: 2.92 (1.62 to 5.24)^d^	
Mortality, Pattakos, 2012 [42]	Cardiac surgery	644	95% versus 89%	0.007
Mortality, Rao, 2004 [43]	ACS	24,112	aHR: 3.94 (3.26 to 4.75)	< 0.05
			RR: 2.60 (2.22 to 3.03)^d^	< 0.05
Mortality, Shehata, 2012 [19]	CABG	2,102	OR: 0.44 (0.32 to 14.1)	NS
Mortality, Shishehbor, 2009 [44]	AMI	3,575	aHR: 3.89 (2.66 to 5.68)	< 0.001
			RR: 1.30 (0.90 to 1.88)^d^	< 0.001
Mortality, Singla, 2007 [45]	AMI	370	RR: 2.36 (1.49 to 3.76)^d^	
Mortality, Wu, 2001, [46]	AMI	78,974	RR: 2.51 (2.42 to 2.61)^d,g^	< 0.05
Mortality, Yang, 2005 [47]	AMI	85,111	aOR: 1.67 (1.48 to 1.88)	< 0.05
			RR: 3.03 (2.85 to 3.21)^d^	< 0.05
Outcomes: myocardial infarction or ischemic events^h^ in adults				
AMI, Jani, 2007 [37]	AMI	4,623	RR: 1.19 (0.82 to 1.74)^d^	
AMI, Jolicœur, 2009 [38]	AMI	5,188	RR: 3.05 (1.85 to 5.04)^d^	
IE^h^, Murphy, 2007 [40]	Cardiac surgery (UK)	8,518	aOR: 3.35 (2.68 to 4.35)^c^	< 0.05
AMI, Nikolsky, 2009 [41]	AMI	2,060	RR: 3.28 (1.44 to 7.49)^d^	
AMI, Pattakos, 2012 [42]	Cardiac surgery	644	2.8% versus 0.31%	< 0.01
AMI + death, Rao, 2004 [43]	ACS	2,401	HR: 3.08 (2.84 to 3.35)^d^	< 0.05
AMI, Shishehbor, 2009 [44]	ACS	3,575	aHR : 3.44	< 0.001
AMI + death, Singla, 2007 [45]	AMI	370	aOR: 2.57 (1.41 to 4.69)	< 0.001
			RR: 2.10 (0.83 to 5.30)^d^	
AMI, Yang, 2005 [47]	AMI	85,111	aOR: 0.95 (0.83 to 1.09)^d^	< 0.05
**Outcomes in children**	**Health problem**	**Children**	**Risk**^**c**^	***P*-value**
LMV (days), 2011 [48]	Cardiac surgery	270	HR: 0.71 (0.54 to 0.92)	0.009
LMV (days), 2013 [49]	Cardiac surgery	335	HR: 2.6 (2.0 to 3.4)	< 0.001
PICU LOS (days), 2013 [49]	Cardiac surgery	335	LOS: 8 ± 0.9 versus 3.5 ± 2	< 0.001
Hospital LOS, 2011 [24]	Cardiac surgery	802	aHR: 0.65 (0.49 to 0.87)	< 0.001
Wound infection, 2010 [50]	Cardiac surgery	216	aOR: 7.87 (1.63 to 37.92)	< 0.001

ACS: acute coronary syndrome; aHR: adjusted HR; AMI: acute myocardial infarction; aOR: adjusted OR; CABG: coronary artery bypass graft; card surg: cardiac surgery; CI: confidence interval; Hct: hematocrit; HR: hazard ratio; IE: ischemic events; LMV: length of MV; LOS: length of stay; MI: myocardial infarction; MV: mechanical ventilation; OR: odds ratio; PICU: pediatric intensive care unit; RBC: red blood cell; NS: not significant; RCT: randomized controlled trial; UK: United Kingdom.

^a^Studies on the relationship between the length of storage of RBC units and outcomes of transfused cardiac patients are excluded from this table.

^b^Year of publication.

^c^Transfused patients versus no RBC transfusion or restricted RBC transfusion strategy.

^d^Chatterjee *et al*. [51] completed a systematic review that included the studies marked by^d^ in this table, plus a small RCT conducted by Cooper *et al*. [52]. Overall, the risk ratio of death in transfused patients versus controls was 2.91 (95% confidence interval (CI): 2.46 to 3.44), but there was a very significant heterogeneity (*I*^*2*^ = 92%).

^e^Pre-transfusion hemoglobin concentration ≤ 80 g/L.

^f^Pre-transfusion hemoglobin concentration > 80 g/L.

^g^The study by Wu *et al*. [46] enrolled patients ≥ 65 years of age with AMI. RBC transfusion was associated with a reduction in 30-day mortality if hematocrit < 24% (aOR = 0.22; 95% CI: 0.11 to 0.45) or between 30% and 33% (aOR = 0.69; 95% CI: 0.53 to 0.89), but the risk of mortality was not increased in patients with a hematocrit > 33% who received RBC transfusion.

^h^Ischemic events: myocardial infarction, stroke, renal impairment, or failure.

Three studies reported some transfusion benefits if the Hb level is low (<80 g/L), but they also reported harm with high Hb level [35,36,46]. A retrospective descriptive epidemiological study of 78,974 patients older than 65 years with acute myocardial infarction showed that RBC transfusion was associated with a lower risk of 30-day mortality if hematocrit was below 24% (odds ratio (OR): 0.22; 95% CI: 0.11 to 0.45) or between 30% and 33% (OR: 0.69; 95% CI: 0.53 to 0.89), but not in cardiac patients with hematocrit above 33% [46]. Alexander *et al*. [35] reported that RBC transfusion tended to have a beneficial impact on mortality if the nadir hematocrit was ≤ 24% (adjusted odds ratio (aOR): 0.68; 95% CI: 0.45 to 1.02), but the opposite was found if it was > 30% (aOR: 3.47; 95% CI: 2.30 to 5.23). Aronson *et al*. [36] reported that RBC transfusion in patients with myocardial infarction decreased the risk of mortality if the pre-transfusion Hb level was ≤ 80 g/L (adjusted hazard ratio (aHR): 0.13: 95% CI: 0.03 to 0.65), but the risk was increased if the Hb level was > 80 g/L (aHR: 2.2; 95% CI: 1.5 to 3.3). Shehata *et al*. [19] reported a lower risk of mortality in 2,102 adults who were transfused after coronary artery bypass graft (CABG), but the association was not statistically significant (adjusted OR (aOR) = 0.44; 95% CI: 0.32 to 14.1).

On the other hand, six studies of adults undergoing cardiac surgery, CABG or with myocardial infarction reported increased mortality in transfused cardiac patients [39,40,42-44,47]. Rao *et al*. [43] published a descriptive epidemiological study on 24,112 patients with acute coronary syndrome who were enrolled in three large randomized controlled trials (RCTs). They compared the outcomes of those who received at least one RBC transfusion (n = 21,711) and those who did not (n = 2,401); RBC transfusion was associated with an increased HR for 30-day mortality (HR = 3.94; 95% CI: 3.26 to 4.75). Probability of 30-day mortality was higher in transfused patients with nadir hematocrit values above 25%. In the systematic review of Chatterjee *et al*. [51], the risk ratio of death in transfused patients versus controls was 2.91 (95% CI: 2.46 to 3.44) and the risk of secondary myocardial infarction was 2.04 (95% CI: 1.06 to 3.93), but there was a very significant heterogeneity in both instances (*I*^*2*^: 92% and 98% respectively). Garfinkle *et al*. [53] also published a systematic review on 11 observational studies that enrolled 290,847 patients with acute coronary syndrome: the unadjusted OR of mortality in transfused patients ranged from 1.9 to 11.2; a meta-analysis was not performed because there was too much heterogeneity, but the data suggested a protective effect of RBC transfusion if nadir Hb drops below 80 g/L and neutral or harmful effects above 110 g/L. In summary, there is evidence in adults with cardiac disease that RBC transfusion is associated with mortality and ischemic events.

### Pediatric cardiac patients

There is less evidence in children. Three studies reported prolonged length of mechanical ventilation in children who received RBC transfusion after cardiac surgery [48,49,54], three reported prolonged length of PICU and/or hospital stay [24,49,54] and three reported an increased incidence of infections [33,50,54]. In 657 consecutive children undergoing open heart surgery, Székely *et al*. [33] found an association between total volume of blood transfusion and the rate of infections (aOR: 1.01; 95% CI: 1.002 to 1.02, *P* < 0.01), but no association with mortality (aOR: 1.00; 95% CI: 0.99 to 1.02, *P* = 0.65).

### Risks/benefits of RBC transfusions: summary

Is it justified to prescribe more RBC transfusions to cardiac than to non-cardiac ICU patients? The available data on this question are inconclusive: while some descriptive studies suggest a benefit to transfusion, most studies associate RBC transfusion to worse outcomes. Descriptive studies cannot prove that there is a cause-effect relationship between a risk factor and a given outcome: adjustment for confounders like volume of RBC transfusion and administration of other blood products can be done, but a possibility always remains that unknown confounders play a role. Moreover, severity of illness is associated with both transfusion and mortality [8,24]. A multivariate analysis cannot deconstruct such confounding by indication [55]; only RCTs can [56].

### Transfusion related adverse events

The safety of blood products with respect to transfusion-transmitted infectious diseases has improved greatly in recent decades. Presently, the greatest concern is non-infectious serious hazards of transfusion (NISHOT) [57-59].

NISHOT may be non-immune or immune-mediated. Short-term non-immune NISHOT include transfusion-related circulatory overload (TACO) and overtransfusion. Overtransfusion is of concern because of the risk of TACO and/or hyperviscosity. Viscous blood flow may impair DO_2_ in small vessels and decrease coronary blood flow. Barr *et al*. [60] reviewed the data of 1,474 transfused patients: 19% of them were overtransfused according to the British RBC transfusion guidelines. Sabatine *et al*. [61] reported an increased mortality in patients with ST-elevation myocardial infarction when Hb value was > 170 g/L (OR = 1.79; 95% CI: 1.18 to 2.71, *P* = 0.007) and > 160 g/L (OR = 1.31; 95% CI: 1.03 to 1.66, *P* = 0.027) in patients with non-ST-elevation myocardial infarction.

Immune-mediated NISHOT include hemolytic and allergic reactions, transfusion-related immunomodulation (TRIM), transfusion-related acute lung injury (TRALI), nosocomial infections, transfusion-associated graft versus host disease, and alloimmunization to RBC and HLA antigens [57,62,63].

In critically ill cardiac patients, TRIM may represent a significant ‘second-hit’ when added to pre-existing organ dysfunction and/or a systemic inflammatory response syndrome (SIRS), which may result in TRALI and multiple organ dysfunction syndrome. Vlaar *et al*. [64] reported that in cardiac surgery patients, RBC transfusion was associated with an increase pulmonary leak index, an early marker of acute lung injury. Inflammatory markers in bronchoalveolar lavage were increased in transfused cardiac surgery patients when compared to controls [65]. TRALI may be an important underdiagnosed cause of lung injury in cardiac and non-cardiac ICU patients [66,67].

Although NISHOT are currently the most important causes of transfusion-related fatalities, their incidence rate in cardiac patients is not well characterized [58].

### Restrictive or liberal RBC transfusion strategy

As illustrated in Figure 1, the relationship between DO_2_ and O_2_ consumption (VO_2_) remains horizontal as long as compensatory mechanisms (increasing cardiac output and O_2_ extraction) are still effective [10,68]. However, VO_2_ drops with DO_2_ below a critical threshold. Anemia decreases DO_2_ and it is unmistakably deleterious below a certain threshold. The critical Hb level threshold below which DO_2_ becomes significantly impaired and transfusion becomes less harmful than persisting anemia is unknown and likely varies depending upon the underlying condition and clinical status of each patient. Anemia is well endured by healthy subjects: acute isovolemic reduction of Hb level down to 50 g/L was hemodynamically well tolerated by 11 resting healthy humans [69], but delayed memory was observed in 31 healthy young volunteers after a similar reduction of Hb [70]. However, cardiac patients may have higher thresholds of critical DO_2_ than healthy adults and target Hb for transfusion might be different (Figures 1 and 2). Moreover, patients with cardiac disease may also be more vulnerable than the general population to adverse effects of transfusion (disturbed rheology [71] and coagulation [65], NISHOT [57], dysfunctional vasoregulation [72], circulatory overload, and so on).

Confronted with the risks of transfusion and a growing body of literature associating RBC transfusion with adverse outcomes, a number of RCTs have now compared the safety of adopting a restrictive versus liberal RBC transfusion strategy (low versus higher threshold Hb) in critically ill patients with cardiac disease (Table 3).

**Table 3:** Restrictive versus liberal red blood cell (RBC) transfusion strategy in cardiac patients: randomized clinical trials

	**Health problem**	**Patients**	**Mortality**^**b**^	
**First author, year**^**a**^		**(n)**	**(95% CI)**	***P*-value**
Adults				
Bracey, 1999 [73]	CABG	428	RR: 0.52 (0.13 to 2.04)	NS
Carson, 2011 [74]	Hip surgery^c^	2,016	ARR: 0.9 (−1.5 to +3.4)	NS
Cooper, 2001 [52]	Myocardial infarction	46	8% versus 5%	1.0
Hajjar, 2012 [23]	Cardiac surgery	502	6% versus 5%	0.93
Hébert, 2001 [75]	ICU cardiac patients	357	22.5% versus 22.7%	1.00
Johnson, 1992 [76]	CABG	38	No difference^d^	NS
Shehata, 2012 [19]	Cardiac surgery	50	16% versus 4%	NS
Pediatric cardiac surgery				
Cholette, 2011 [77]	Cyanotic	60	1 death	NS
de Gast-Bakker, 2013 [78]	Non-cyanotic	107	No death	NS
Willems, 2010 [79]	Non-cyanotic	125	12.7% versus 6.5%	0.36

ARR: absolute risk reduction; CABG: coronary artery bypass graft; CI: confidence interval; RBC: red blood cell; NS: not statistically significant.

^a^Year of publication.

^b^Restrictive versus liberal transfusion strategy.

^c^Hip surgery in patients older than 50 years with atherosclerosis.

^d^No significant difference in duration or degree of exercise was demonstrated between the two groups.

### RBC transfusion strategy in cardiac adults

We found seven RCTs conducted in adults with cardiac illness [19,23,52,73-76]; all enrolled only hemodynamically stable patients. All but one [23] reported that a restrictive strategy was as safe or safer than a liberal strategy. Three RCTs included more than 50 patients.

In 1999, Hébert *et al*. [80] published the ‘Transfusion Requirements in Critical Care’ (TRICC) study. Euvolemic patients were randomized to receive a RBC transfusion if Hb level was below 100 g/L (liberal strategy) or below 70 g/L (restrictive strategy). Patients with acute myocardial infarction and unstable angina were excluded. A subgroup analysis of 357 patients with cardiovascular disease was published in 2001 [75]; the 30-day all-cause mortality was similar (22.5% versus 22.7%, *P* = 1.00).

Hajjar *et al*. [23] published a single center non-inferiority RCT of 502 adults undergoing cardiac surgery who were allocated to receive a transfusion if hematocrit was below 24% or 30%. The pre-transfusion Hb level was 91 in the restrictive and 105 g/L in the liberal group. They found a similar incidence of 30-day all-cause mortality (6% in the restrictive and 5% in the liberal group, *P* = 0.93), cardiogenic shock (5% versus 9%, *P* = 0.42), acute respiratory distress syndrome (1% versus 2%, *P* = 0.99) and acute renal failure (5% versus 4%, *P* = 0.99). The number of RBC units transfused was independently associated with 30-day all-cause mortality (HR = 1.2/unit; 95% CI: 1.1 to 1.4, *P* = 0.002).

Carson *et al*. [74] published the ‘Functional Outcomes in Cardiovascular Patients Undergoing Surgical Hip Fracture Repair’ (FOCUS) study, a large multicenter RCT of 2,016 adults over 50 years of age with known atherosclerotic disease undergoing a hip surgery and who had a Hb level below 100 g/L within 3 days post-surgery. The restrictive group was transfused if Hb level fell below 80 g/L, the liberal group if below 100 g/L. Primary outcome was ability to walk 10 feet unassisted or death 60 days post-randomization. No difference was found in any of the outcomes, including survival, postoperative complications, activities of daily living and disposition.

In summary, the available RCTs suggest that a restrictive transfusion strategy (threshold Hb level for transfusion: 70 or 80 g/L) appears as safe as a liberal strategy in stable adult ICU patients with cardiac disease.

### RBC transfusion strategy in cardiac children

Three RCTs compared a restrictive and a liberal transfusion strategy after pediatric cardiac surgery.

In the ‘Transfusion Requirements In PICU’ (TRIPICU) study [81], 637 stabilized critically ill children were randomized to receive a RBC transfusion if their Hb dropped below 70 or 95 g/L [81]. Patients were considered stable if mean systemic arterial pressure was not less than two standard deviations below the normal mean for age and if cardiovascular treatment (fluids and/or medication) had not been increased for at least two hours before enrollment. Children with cyanotic cardiac disease and neonates under 28 days were excluded. A sub-group analysis of 125 cardiac children enrolled in TRIPICU demonstrated no significant difference in new or progressive multiple organ dysfunction syndrome (restrictive versus liberal group: 12.7% versus 6.5%, *P* = 0.36), PICU length of stay (7.0 ± 5.0 versus 7.4 ± 6.4 days) or 28-day mortality (3.2% versus 3.2%) [79].

An RCT compared the outcome of cardiac children older than 6 weeks randomized to receive a RBC transfusion if Hb level dropped below 80 g/L or 108 g/L [78]. Patients with cyanotic cardiac disease were excluded. Randomization occurred before surgery; the research protocol with respect to RBC transfusion was initiated in the operating room and maintained up to PICU discharge. One hundred patients were enrolled and retained for analysis. Duration of mechanical ventilation, length of PICU stay and incidence of adverse events were similar in both groups, but length of hospital stay was shorter in the restrictive group (median: 8 {interquartile range: 7 to 11} versus 9 {7 to 14} days, *P* = 0.063).

Physicians target higher Hb values for critically ill children with cyanotic heart disease [28,29]. In an RCT completed by Cholette *et al*. [77], 60 children were randomized after Glenn or Fontan palliation to a restrictive (transfusion if Hb < 90 g/L) or liberal group (<130 g/L). One death was observed (liberal group). There was no difference in mean and peak arterial lactate, arterio-venous and arterio-cerebral oxygen content. Although not powered to demonstrate statistical significance, mortality, PICU and hospital length of stay, duration of mechanical ventilation, dose and duration of inotropic support were similar.

### Goal-directed transfusion therapy

Goal-directed RBC transfusion therapy targeting a physiological goal may be a more appropriate target than specific Hb levels. The concept of goal-directed therapy is well illustrated by the RCT conducted by Rivers *et al*. [82]: 266 adults with severe sepsis or septic shock were randomized to be monitored or not with ScvO_2_. Patients allocated to ScvO_2_ monitoring were also assigned to a bundle of treatment to maintain their ScvO_2_ over 70%; this bundle included, in sequential order, mechanical ventilation, fluid bolus, vasoactive drugs and RBC transfusion if the ScvO_2_ remained under 70% after all other interventions. Mortality was 30.5% with goal-directed therapy versus 46.5% in controls.

There are currently no hard data on goal-directed transfusion therapy in cardiac patients. Goals that can be considered include physiologic parameters like DO_2_ measured by a Swan-Ganz catheter or locally with near infrared spectroscopy (NIRS), global oxygen consumption (VO_2_), blood lactate, mixed venous O_2_ saturation (Sv’O_2_), ScvO_2_, tissue O_2_ saturation and O_2_ extraction rate [10]. Well-conducted research is required to demonstrate the reliability and clinical applicability of a test before we start to use it at the bedside. There are examples of tests that were too rapidly adopted by the medical community. For example, regional (splanchnic and/or renal) NIRS is frequently used to detect low cardiac output in ICU patients; a recent study showed that its positive predictive value is so low that it cannot be considered a reliable test [83]. Repeated (‘dynamic’) measurements of NIRS might be more informative than ‘static’ measures [84], but this remains to be proven. Transfusion therapy guided by ScvO_2_ is another candidate. The results of the trial by Rivers *et al*. [82] suggested that RBC transfusion might be useful in septic patients. Another RCT conducted in 102 children with severe sepsis or fluid refractory shock reported similar results (mortality: 11.8% versus 39.2% in controls) [85]. However, in the ProCESS three arms RCT [86], 1,341 adults were allocated to a protocol-based goal-directed therapy that required the placement of a central venous catheter, a protocol-based standard therapy without such catheter, or usual care; no difference was observed in any outcomes, which questions the results of the RCTs by Rivers and de Oliveira [82,85]. Moreover, the specific contribution of RBC transfusions was unclear in these RCTs and none enrolled cardiac patients.

Currently, we do not know what physiologic parameters we must use to guide a goal-directed transfusion therapy in cardiac patients. Further clinical studies are required before a given goal that would help practitioners to precisely decide when to transfuse cardiac patients can be recommended in this population.

## Conclusion

Anemia is common in patients with cardiac disease and is associated with mortality and morbidity. RBC transfusion is the best way to rapidly increase the Hb level, but it is not risk-free: storage lesion and NISHOT, including prothrombotic and proinflammatory effects, may cause transfusion-related adverse events.

The Hb level at which the risk of anemia outweighs the risk of transfusion is not well known. Three observational studies suggest that there might be some benefit to give a RBC transfusion to adults with acute coronary syndrome if their Hb level is < 80 g/L, but many more studies suggest that RBC transfusion might be harmful in this population if their Hb level is higher than 80 to 100 g/L. The question of RBC transfusion to adults with acute coronary syndrome is not addressed in the guidelines of the American College of Cardiology, the American Heart Association and the Canadian Cardiovascular Society [87]. However, a number of RCTs in hemodynamically stable cardiac patients suggest a level of Hb (about 80 g/L) above which it appears safe not to transfuse, thereby avoiding the risks related to transfusions.

Data in children are scarce. In stable acyanotic cardiac children, a Hb level above 70 or 80 g/L appears to be well tolerated without RBC transfusion. In children with cyanotic heart lesions, Hb level over 90 g/L appears safe. The threshold Hb level for unstable cardiac children and for neonates is unknown. Goal-directed transfusion therapy is a promising avenue for future research.

Severity of illness, anemia and transfusions are interconnected with outcome in cardiac patients, and their individual contribution to outcome remains unresolved. Further RCTs are necessary to disentangle this relationship.

## Abbreviations

ACS: acute coronary syndrome; aHR: adjusted HR; AMI: acute myocardial infarction; aOR: adjusted OR; APACHE: acute physiology and chronic health; CABG: coronary artery bypass graft; CI: confidence interval; DO_2_: O_2_ delivery; Hb: hemoglobin; Hct: hematocrit; ICU: intensive care unit; IE: ischemic events; HR: hazard ratio; MI: myocardial infarction; MODS: multiple organ dysfunction syndrome; MV: mechanical ventilation; NIRS: near infrared spectroscopy; NISHOT: non-infectious serious hazard of transfusion; OR: odds ratio; PCI: percutaneous coronary intervention; PICU: pediatric ICU; PRISM: pediatric risk of mortality; RBC: red blood cell; RCT: randomized controlled trial; RR: relative risk; ScvO_2_: central venous O_2_ saturation; Sv’O_2_: mixed venous O_2_ saturation; TACO: transfusion-associated circulatory overload; TRALI: transfusion-related acute lung injury; TRIM: transfusion-related immunomodulation; VO_2_: O_2_ consumption.

## Competing interest

The authors declare that they have no competing interests.

## Authors’ contribution

GD, KH and JL wrote, revised and approved the manuscript in its final form.
